# Multimodal imaging brain connectivity analysis (MIBCA) toolbox: preliminary application to Alzheimer’s disease

**DOI:** 10.1186/2197-7364-1-S1-A61

**Published:** 2014-07-29

**Authors:** Andre Santos Ribeiro, Luís Miguel Lacerda, Nuno André da Silva, Hugo Alexandre Ferreira

**Affiliations:** Center for Neuropsychopharmacology, Division of Brain Sciences, Department of Medicine, Imperial College London, London, UK; Institute of Biophysics and Biomedical Engineering, Faculty of Sciences of the University of Lisbon, Lisbon, Portugal; Centre for Neuroimaging Sciences, Institute of Psychiatry, King’s College London, London, Denmark Hill, UK; Institute of Neuroscience and Medicine-4, Forschungszentrum Juelich GmbH, Juelich, Germany

The Multimodal Imaging Brain Connectivity Analysis (MIBCA) toolbox is a fully automated all-in-one connectivity analysis toolbox that offers both pre-processing, connectivity, and graph theory analysis of multimodal images such as anatomical, diffusion, and functional MRI, and PET [1]. In this work, the MIBCA functionalities were used to study Alzheimer’s Disease in a multimodal MR/PET approach.

Data from 11 healthy subjects and 10 AD patients were obtained from the Alzheimer’s Disease Neuroimaging Initiative (ADNI) database (adni.loni.usc.edu), including T1-weighted (T1w), Diffusion Tensor Imaging (DTI) data, and ^18^F-AV-45 (florbetapir) dynamic PET data from 40-60 min post injection (4x5min). Both MR and PET data were automatically pre-processed for all subjects using MIBCA. The T1w data was parcellated into cortical and subcortical regions-of-interest (ROIs), and the corresponding thicknesses and volumes were calculated. DTI data was used to compute structural connectivity matrices based on fibers connecting pairs of ROIs. Lastly, dynamic PET images were summed, and the Standard Uptake Values calculated for each ROI.

**Figure 1:**
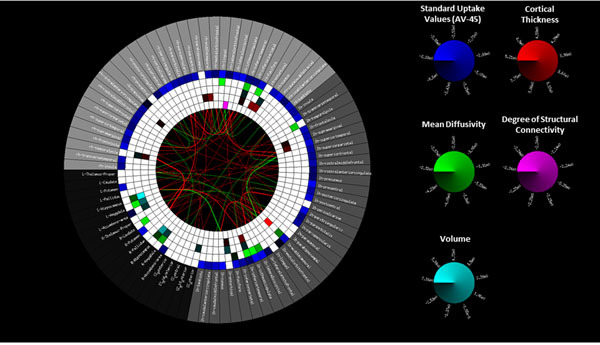
Group difference Connectrogram. Significant differences between Healthy Subjects and AD patients are highlighted in color in the inner rings. From outside to inside rings: region-of-interest (ROI), Standard Uptake Value (18F-AV-45), mean diffusivity, ROI volume, cortical thickness, node degree computed from structural connectivity.

**Figure 2:**
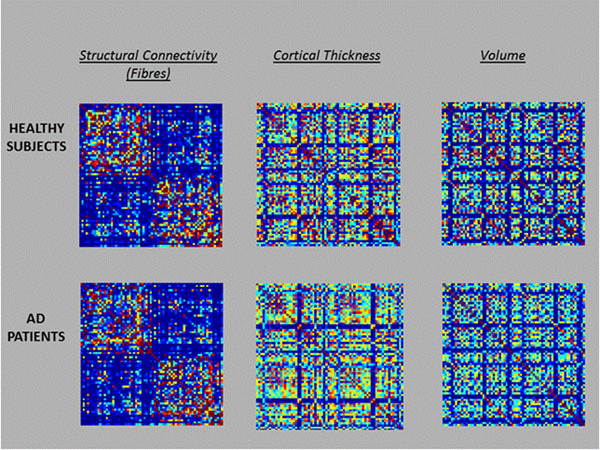
Group connectivity matrices for Healthy Subjects (upper row), and AD patients (lower row). From left to right are represented the structural, cortical thickness and cortical volume connectivity matrices.

An overall higher uptake of ^18^F-AV-45, consistent with an increased deposition of amyloid-Beta, was observed for the AD group. Additionally, patients showed significant cortical atrophy (thickness and volume) especially in the enthorhinal and temporal areas, and a significant increase in Mean Diffusivity (MD) in the hippocampus, amygdala and temporal areas, Figure 1. Furthermore, patients showed an overall decrease of both inter- and intra- hemispherical structural connections (tracts), Figure 2. Finally, the 3D-graph visualization showed that the structural loss was global and asymmetric, Figure 3.

This work shows the potential of the MIBCA toolbox for the study of AD, as findings were shown to be in agreement with the literature [2–4]. Here, only structural changes and beta amyloid accumulation were considered. Yet, MIBCA is further able to process fMRI and different radiotracers, and combine all the information in order to provide new insights into AD.
